# Temporal Resolution Needed for Auditory Communication: Measurement With Mosaic Speech

**DOI:** 10.3389/fnhum.2018.00149

**Published:** 2018-04-24

**Authors:** Yoshitaka Nakajima, Mizuki Matsuda, Kazuo Ueda, Gerard B. Remijn

**Affiliations:** ^1^Department of Human Science, Faculty of Design/Research Center for Applied Perceptual Science, Kyushu University, Fukuoka, Japan; ^2^Nihon Kohden Corporation, Tokyo, Japan

**Keywords:** speech, spectro-temporal resolution, intelligibility, mosaic, movie frames

## Abstract

Temporal resolution needed for Japanese speech communication was measured. A new experimental paradigm that can reflect the spectro-temporal resolution necessary for healthy listeners to perceive speech is introduced. As a first step, we report listeners' intelligibility scores of Japanese speech with a systematically degraded temporal resolution, so-called “mosaic speech”: speech mosaicized in the coordinates of time and frequency. The results of two experiments show that mosaic speech cut into short static segments was almost perfectly intelligible with a temporal resolution of 40 ms or finer. Intelligibility dropped for a temporal resolution of 80 ms, but was still around 50%-correct level. The data are in line with previous results showing that speech signals separated into short temporal segments of <100 ms can be remarkably robust in terms of linguistic-content perception against drastic manipulations in each segment, such as partial signal omission or temporal reversal. The human perceptual system thus can extract meaning from unexpectedly rough temporal information in speech. The process resembles that of the visual system stringing together static movie frames of ~40 ms into vivid motion.

## 1. Introduction

Speech can remain considerably intelligible even when it is drastically manipulated in the temporal domain. One example is the word intelligibility (articulation) of speech in which 50-ms portions are alternately played and silenced, as reported by Miller and Licklider (1950), who discovered the illusory continuity (see also Vicario, 1960). Intelligibility does not change in such “gated speech” even if the temporal gaps are simply removed, shortening the total duration (Fairbanks and Kodman, 1957; Shafiro et al., 2016). The perception of locally time-reversed speech is also to be noted. When speech is cut into segments of 50 ms, for example, and if each segment is reversed in time, intelligibility is still quite well preserved (Steffen and Werani, 1994; Saberi and Perrott, 1999; Ueda et al., 2017).

Neuroscientific research performed in the last decade has suggested that different types of neural oscillations are involved in the segmentation and organization of speech into perceptual units (Giraud and Poeppel, 2012; Chait et al., 2015). Neural oscillations at modulation frequencies around 30–50 Hz, corresponding to a temporal resolution around 20–33 ms, are considered to be involved in phonemic processing. If this temporal resolution is attained, the human auditory system should receive sufficient information to grasp the rhythmic intensity fluctuations in speech and music (Ding et al., 2017).

**Figure 1:**
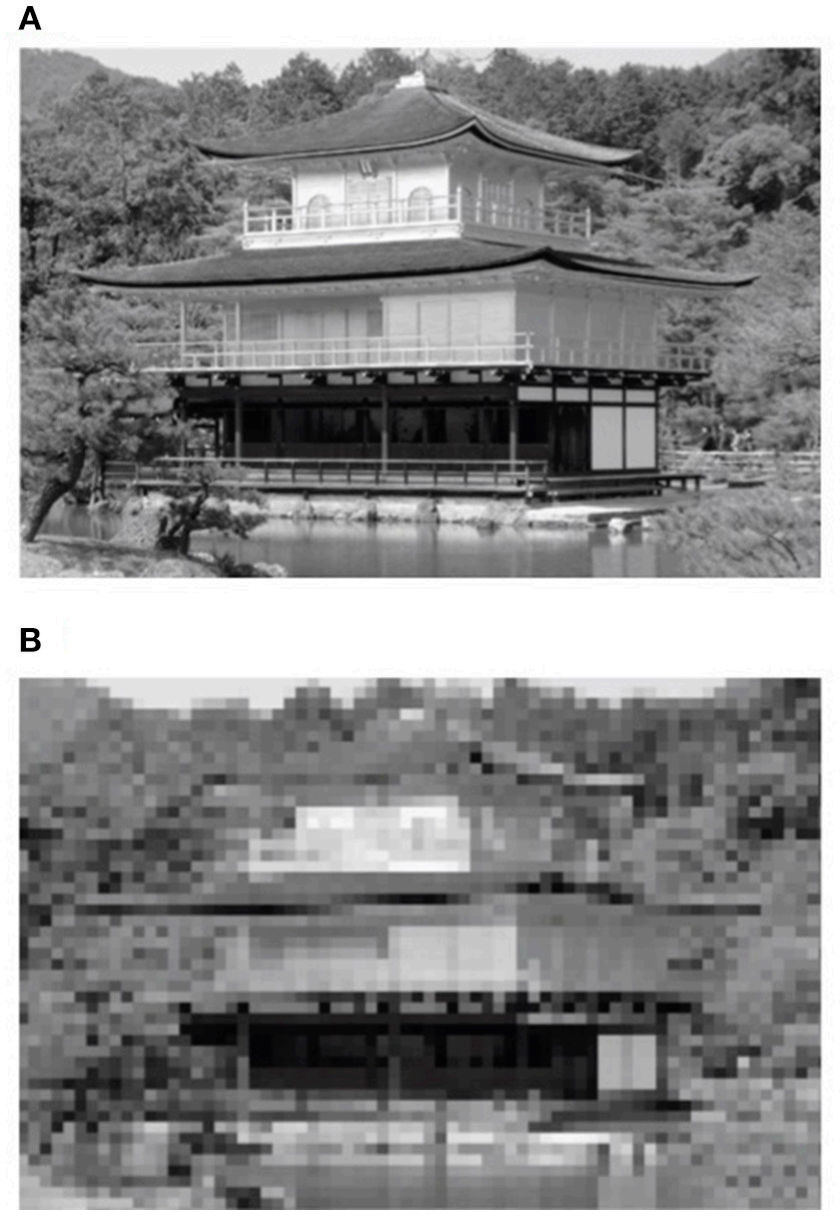
Original (**A**; taken by Feng Li) and mosaic image **(B)** of the Kinkakuji, Kyoto, Japan.

To some extent, the segmentation and organization process of speech into perceptual units resembles that of how the visual system strings together static movie frames into motion pictures. The motion of visual objects can be expressed vividly by presenting successive still frames in a ratio of 24 frames per second: A temporal resolution of 42 ms is sufficient to perceive motion pictures. This brought our research question: Is it possible to express speech sentences utilizing auditory counterparts of movie frames? One of the ways to deal with this issue is to use locally time-reversed speech (Saberi and Perrott, 1999). This paradigm is used widely to measure the temporal resolution needed for speech communication (Ueda et al., 2017). Basically, this procedure is considered to make the temporal information within each segment unavailable. Thus, each segment plays the role of a movie frame. This analogy does not seem to work precisely, however, since the reversed segment still keeps the original temporal change in the reversed direction. A very short explosion, for example, may be preserved as a similar noise burst in a reversed segment, but it may appear in a distorted timing. This can either improve or reduce the listeners' performance. In other words, an experimental noise, whose influence is unpredictable even qualitatively, is left. We thus created “mosaic speech” in analogy with visual mosaic images (Harmon, 1973) of monochrome pictures (Figure 1). This was done by concatenating local spectra of speech signals, which by definition were static, resulting in a new type of degraded speech suitable to study the temporal resolution needed for speech perception. Since each local spectrum in the signal should be playable as a steady-state sound, we added up 17 narrow-band noises, whose frequency bands covered a range 0.1–4.4 kHz, corresponding to critical bands simulating the auditory periphery (Fastl and Zwicker, 2007).

The past paradigm to utilize locally time-reversed speech has played an important role to shed light on the issue of temporal resolution needed for speech communication. As mentioned above, however this paradigm contains unavoidable experimental contamination for that purpose, since the temporal fine structure of each speech segment is preserved but reversed; the fine structure is not suppressed.

The preserved temporal fine structure may inappropriately facilitate speech perception. For example, stop consonants often begin with explosions, approximately very short band-noise bursts, and this is one of the cues to identify stop consonants (Liberman, 1996). If an explosion is located in the middle of a segment, a short portion of band noise is still there even when the segment is reversed in time, and this may help the perception of the stop consonant. The locally time-reversed speech was not able to suppress the fine temporal structure to be excluded in this case.

On the other hand, the reversing procedure may have a negative influence on speech perception. Stop consonants are also differentiated perceptually by formant transitions just before the following vowel, i.e., ascending or descending formant frequencies (Liberman, 1996). If a clear formant transition is included in a temporal segment, then the ascending or descending movement is reversed in time by the time-reversing procedure; this is very likely to distort consonant identification.

Thus, locally time-reversed speech patterns are not necessarily very suitable to control temporal resolution systematically. The unchanged, although reversed, waveforms may preserve temporal information that should not be used by the participants, or the reversing procedure may sneak unrelated disturbance to the experiment. In order to avoid this kind of experimental contamination, the only practical way is to replace each speech segment with a sound without a clear temporal structure keeping the basic spectral shape. This was the most important reason we created the mosaic speech paradigm. Once the paradigm is established, it can be employed for many other purposes just as the mosaic paradigm in vision.

It is important to compare experimental results obtained in the locally time-reversing paradigm and in the mosaic speech paradigm. If there is a discrepancy, perhaps the past experiments in the locally time-reversed paradigm should be interpreted with some caution.

Speech signals can be mosaicized in the coordinates of time and frequency. By manipulating how fine or rough the time-frequency mosaic tiles are, we are able to generate various mosaic speech stimuli, within the constraint of the time-frequency uncertainty principle (Cohen, 1989). This can be done without adding or changing irrelevant cues, making it possible to create standardized tests to check the temporal or the frequency resolution given to or needed by the auditory system for speech perception. In the present study, we mainly investigated the precision of temporal resolution (see also Supplementary Material). The frequency resolution was fixed at that of critical bandwidths, and the temporal (time) resolution was varied systematically. In order to obtain behavioral data on the temporal resolution needed for speech perception, we measured the intelligibility of Japanese mosaic speech. Mosaic speech neither contains distinct pitch information, nor cues as to the temporal fine structures of the original speech.

**Figure 2:**
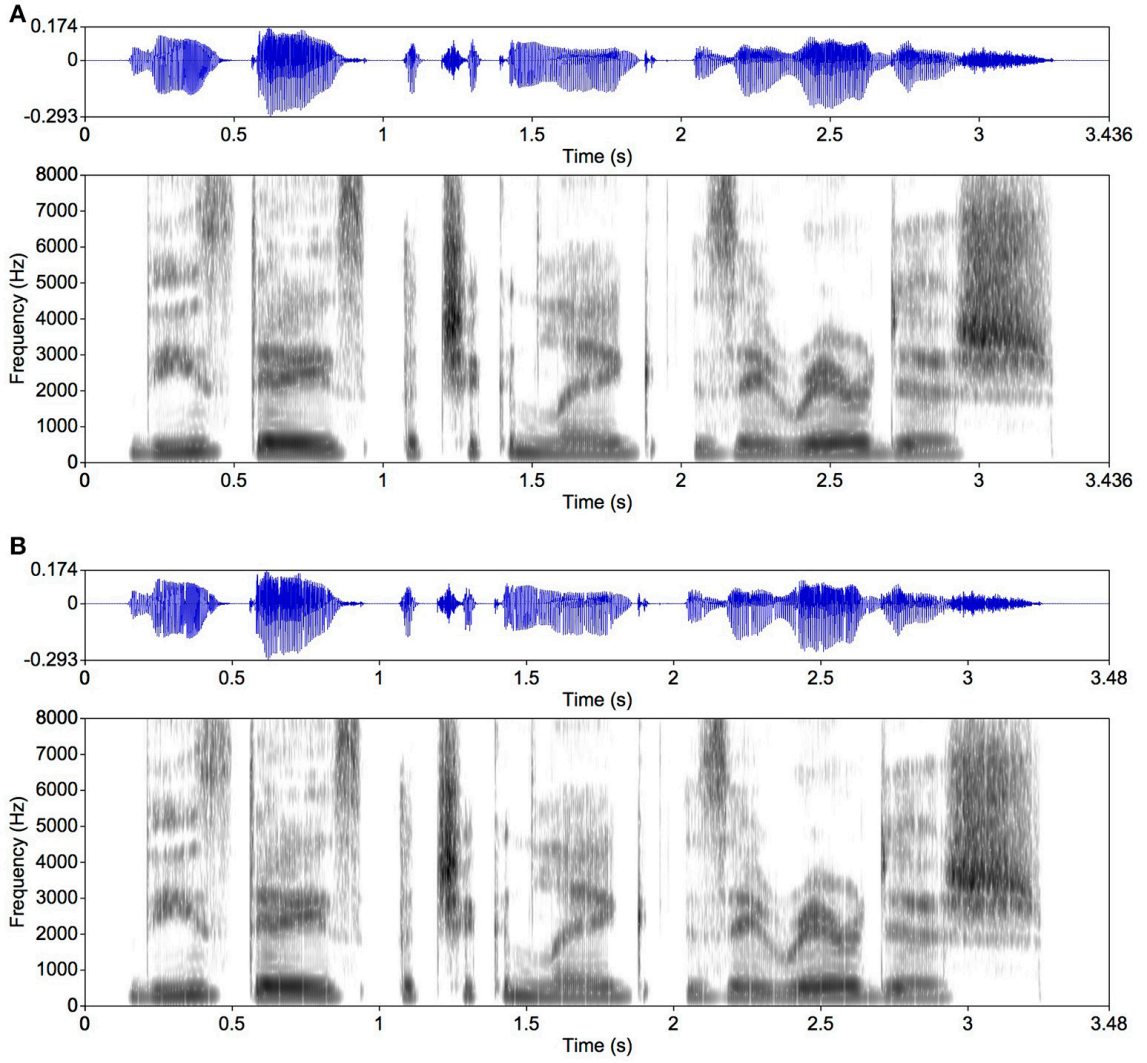
An example of original speech. Speech waveforms and spectrograms are presented. The sound energy distribution in each spectrogram is indicated by the gray density (the darker the gray, the higher the sound-energy density). **(A)** An English sentence (for illustration) as original speech spoken by a female native speaker, saying “These days a chicken leg is a rare dish.” **(B)** The original speech divided into 80-ms segments smoothed with 5-ms rise and fall times. The white vertical lines in the spectrogram in **(B)** are the rise and fall times that delimit the speech segments. No such vertical lines are observed in the spectrogram in **(A)**. The original speech data were taken from the NTT-AT Multi-Lingual Speech Database 2002. Figures 2–4 were made with Praat (Boersma and Weenink, 2016).

**Figure 3:**
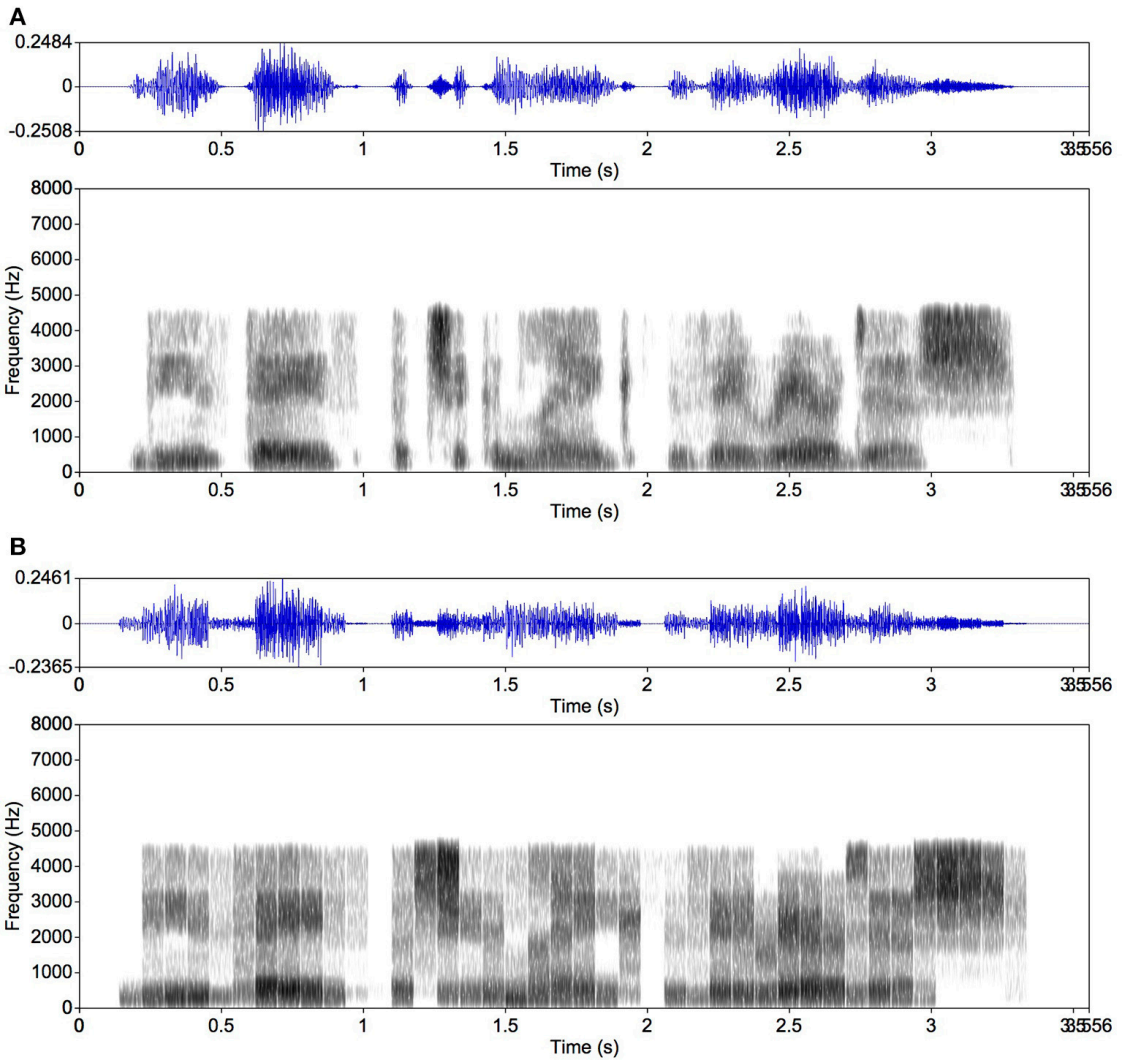
Examples of noise-vocoded speech (Shannon et al., 1995; Smith et al., 2002; Ellermeier et al., 2015; Kishida et al., 2016) and mosaic speech. The same original speech as in Figure 2 was used. **(A)** Noise-vocoded speech made of amplitude-modulated noises in 17 critical bands; **(B)** mosaic speech: the noise-vocoded speech mosaicized into 80-ms segments. Intuitively, the mosaic speech was constructed by cutting the original speech as appeared in the spectrogram into time-by-frequency blocks of 80 ms and one critical bandwidth, and by leveling the sound-energy density in each block. Because the uncertainty principle between time and frequency does not allow the spectrum of a noise portion to be kept within a narrow frequency band if the portion appears and disappears abruptly, each block was shaped with a rise time and a fall time of 5 ms.

**Figure 4:**
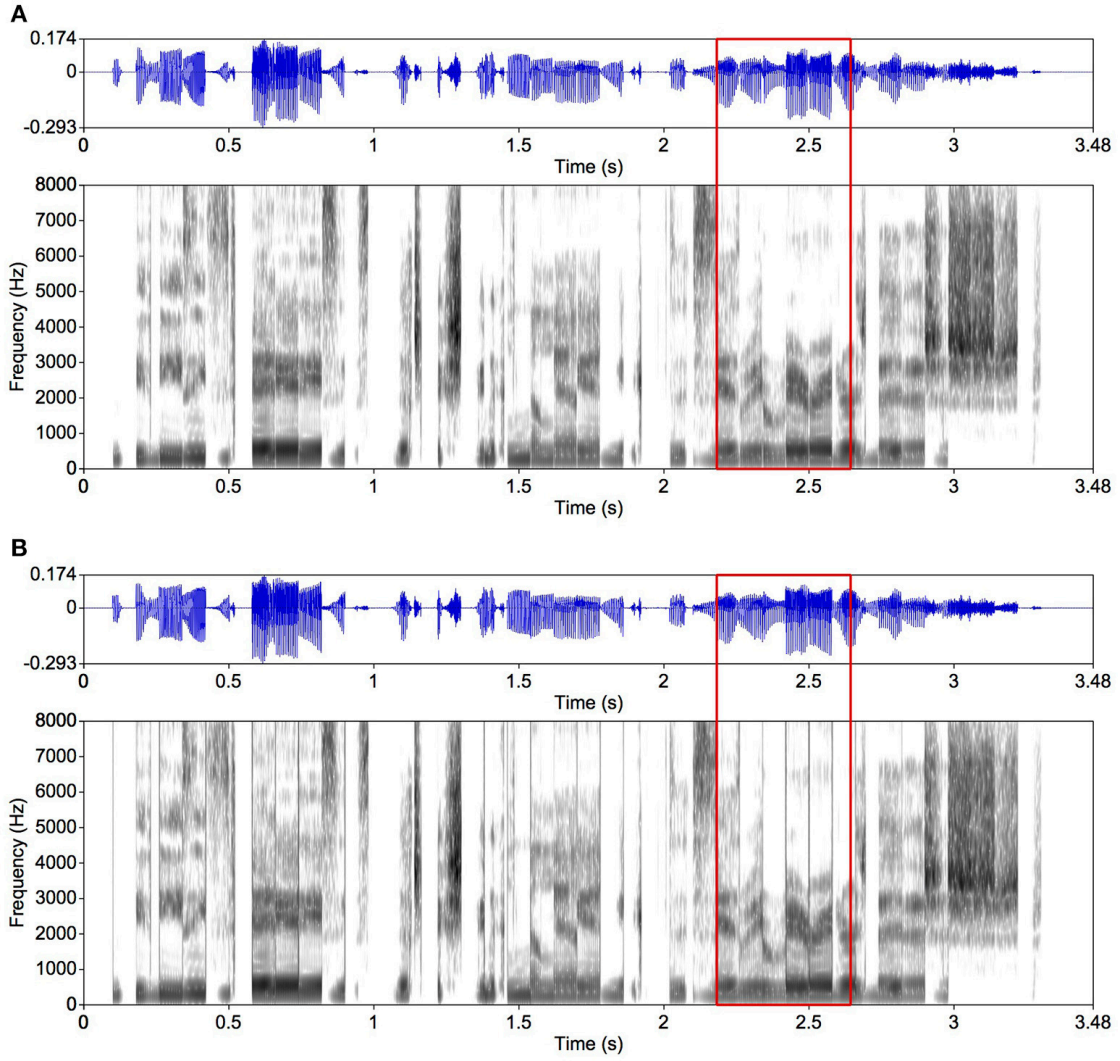
Examples of locally time-reversed speech. The same original speech as in Figure 2 was used. The segment duration was 80 ms. **(A)** Each segment is smoothed with 5-ms rise and fall times; **(B)** another version without smoothing. When focusing on the area in the red frames, vertical gray lines in the spectrogram are observed in **(B)** showing spectral splatters caused by the abrupt onsets and offsets. Such vertical lines are not observed in the spectrogram in **(A)** where the segments are smoothed.

In detail, mosaic speech was made as follows; relevant speech signals are illustrated in Figures 2–**4**. Note that Japanese speech was used in the present experiments, but an English sentence is used in this explanation to enable the reader to see the correspondence between the original speech and its spectrogram. The time axis and the frequency axis of a sound spectrogram are not completely independent of each other, contrary to the horizontal and the vertical axis of visual images. For acoustic signals, the uncertainty principle between time and frequency (the inverse of time) makes it essentially impossible to control both time and frequency very accurately (Cohen, 1989). Since our direct purpose was to gain insight into the temporal aspects of speech communication, we put our priority on obtaining a temporal resolution of 20 ms, the inverse of 50 Hz, considering the fact that a period of vocal-folds vibration of male speakers can be around 10 ms (Raphael et al., 2011). Fortunately, this was still compatible with the finest frequency resolution of the critical bandwidth, i.e., 100 Hz (Fastl and Zwicker, 2007). The smallest possible size of the temporal segmentation for mosaicization was thus determined as 20 ms.

For generating mosaic speech, we first separated the speech signal (Figure 2A) into critical bands. Each critical band contains a temporal intensity fluctuation presumably conveying linguistic information. We generated a band noise in each critical band, which was amplitude-modulated to make its intensity fluctuation equivalent to that observed in the same frequency band of the original speech signal. This follows basically the procedure to make noise-vocoded speech (Shannon et al., 1995; Smith et al., 2002; Kishida et al., 2016), which is exemplified in Figure 3A. This noise-vocoded speech was almost perfectly intelligible (see Ellermeier et al., 2015, for related data); it contains linguistic information sufficient for speech perception. We calculated the intensity fluctuation of the speech signal in each critical band, which enabled us to calculate average intensity in any given temporal segment. We then cut the intensity fluctuation for each critical band into segments of 80 ms, for example, and calculated the average intensity in each segment. By replacing each temporal segment of each critical band with a band noise portion of the same average intensity, mosaic speech was obtained. Each noise portion was smoothed with a rise and a fall time of 5 ms to avoid spectral splatters (Figure 3B).

For one of the intelligibility experiments (Experiment 2), we used not only mosaic speech, but also locally time-reversed speech with smoothing and without smoothing, as well as the original speech. Locally time-reversed speech was made by reversing each segment in time as in Figure 4A (Steffen and Werani, 1994; Saberi and Perrott, 1999). This is a well-established way to degrade the temporal resolution of speech (Ueda et al., 2017). Without smoothing, the abrupt edges of the segments are heard clearly as click-like sounds as can be seen in the spectrogram (Figure 4B). We also prepared the original speech with the same rise and fall times for control (Figure 2B).

## 2. Results

**Figure 5:**
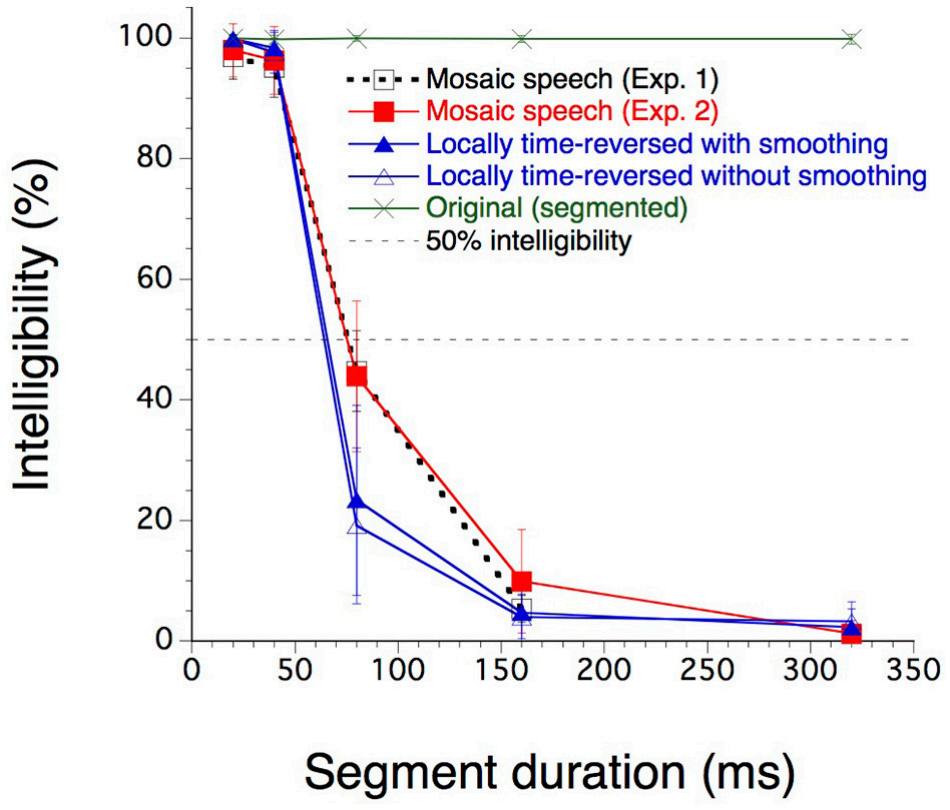
Mora identification scores (%) for the four stimulus conditions (*n* = 4 in Experiment 1 and *n* = 20 in Experiment 2). The dashed horizontal line indicates the 50% level of mora identification. The error bars indicate the standard deviations. Twenty-four participants were asked to write down what they heard in Japanese hiragana letters, indicating morae–based phonological units of Japanese, which are in many cases equal to and sometimes shorter than syllables.

Twenty-four participants (*n* = 4 in Experiment 1 and *n* = 20 in Experiment 2) were asked to write down what they heard in Japanese *hiragana* letters, each corresponding clearly in most cases to one mora—basic phonological units of Japanese, which are in many cases equal to and sometimes shorter than syllables. The percentage of correct mora identification was calculated by counting the number of written morae that corresponded to the morae pronounced in the three sentences assigned to each stimulus condition (Figure 5).

The intelligibility of original speech, as measured as the percentage of correct mora identification, was almost perfect for any segment duration. The intelligibility of mosaic speech and locally time-reversed speech, with or without rise and fall times, was nearly perfect when the segment duration was 20 or 40 ms. Intelligibility decreased monotonically after that as the segment duration increased. The results of Experiment 1 were very close to the results obtained in the same conditions in Experiment 2; Experiment 1 may therefore be considered a kind of pilot experiment whose results were fully replicated in Experiment 2. Thus, only the results of Experiment 2 were statistically analyzed. For each participant and for each stimulus type, except for original speech, the segment duration corresponding to the 50% correct mora identification was calculated by linear interpolation. The average values were 75.3 ms for mosaic speech, 65.8 ms for locally time-reversed speech with rise and fall times, and 64.3 ms for locally time-reversed speech without rise and fall times.

A Friedman two-way analysis of variance by ranks (Siegel and Castellan, 1988) was performed on these 50% points for these three stimulus types. The effect of the stimulus types was significant (*N* = 20, *k* = 3, *F*_*r*_ = 17.5; *p* < 0.001). Multiple comparisons after that (following Siegel and Castellan) indicated significant differences between mosaic speech and locally time-reversed speech with/without rise and fall times (*p* < 0.01; *p* < 0.01). The difference between the two types of locally time-reversed speech was not significant. In sum, the participants' performance was significantly better for mosaic speech than for locally time-reversed speech, and the 50% threshold for Japanese mosaic speech exceeded 70 ms.

## 3. Discussion

For all stimulus conditions, mora identification was nearly perfect for temporal windows as fine as 20 or 40 ms. It dropped sharply, however, except in the original-speech condition, as the temporal windows widened from 40 to 80 ms and further. Mora identification dropped to 10% or below with even wider temporal windows.

### 3.1. Locally time-and-frequency-reversed stimuli

As mentioned earlier, to measure something related to the temporal resolution of the auditory system or acoustic signals is often difficult because time and frequency are not completely independent. In a preliminary study, we reversed speech not only in time but also in frequency, thus imposing a temporal and frequency grid upon Japanese spoken sentences (see Supplementary Material). These locally time-and-frequency-reversed stimuli generated results similar to the present results. Intelligibility dropped sharply when the temporal window widened from 40 to 80 ms.

### 3.2. Mosaic vs. locally time-reversed speech

The results of the experiments showed that the participants' performance was significantly better for mosaic speech than for locally time-reversed speech, either with or without smoothing. This indicates that the perception of locally time-reversed speech was degraded by the reversed temporal fine structure, which itself can be a very interesting research topic in future. In order to measure the temporal resolution needed to make speech signals intelligible, in other words, to measure how far the temporal resolution can be lowered without harming intelligibility, mosaic speech seems more suitable than locally time-reversed speech. Another advantage of mosaic speech is that both the temporal and the frequency resolution can be manipulated for future studies including clinical ones.

### 3.3. Temporal resolution of the auditory system and speech perception

The problem of the time-frequency uncertainty principle is usually more obvious when very short time intervals are concerned. Some experimental paradigms have been employed to circumvent this problem, e.g., phase detection or temporal gap detection. Based on research with these paradigms, the ultimate temporal acuity in the auditory modality, except for dichotic situations, is close to 2 ms (Eddins and Green, 1995). There are often temporal changes in speech taking place in periods around 40 ms. Examples of these changes are formant transitions as in /w + (vowel)/ and /p + (vowel)/ or explosions as in /p + (vowel)/ and /g + (vowel)/, followed by a far weaker intensity. Therefore, it is surprising that a temporal resolution comparable to that of the motion picture system is sufficient to perceive the linguistic content of speech.

One way to assess temporal acuity is to measure the gap detection threshold. This paradigm is used, for example, to investigate decreased speech intelligibility in the elderly with otherwise preserved pure tone thresholds (Ozmeral et al., 2016). Along this line, it would be also necessary to measure the listeners' capacity to grasp linguistic contents even when the temporal resolution of the speech signals is limited, as in a reverberant room. The “mosaic speech” paradigm introduced in the present study can be very useful for this purpose.

The present study shows that linguistic information is conveyed almost perfectly by auditory blocks around 40 ms. It is interesting that linguistic contents of speech can be conveyed almost perfectly by presenting successive spectra at intervals of 40 ms just as the movie system can represent motions by presenting successive static pictures at similar intervals. Although the auditory and the visual modality are different in their peripheries, their cortical organizations may employ similar temporal grids (deCharms et al., 1998).

The present behavioral data corroborate neuroscientific research indicating that neural oscillations around 30–50 Hz are involved in the segmentation and organization of ongoing speech signals into perceptual units (Giraud and Poeppel, 2012; Chait et al., 2015). If phonemic processing as related to formant transitions or noise-vowel transitions is really based on such oscillations, processing should deteriorate for degraded speech whose temporal segment size exceeds ~20–33 ms. This agrees with the present data.

Widening the temporal windows from 40 to 80 ms caused a considerable drop of mora identification both for mosaic speech and locally time-reversed speech. We thus conclude that the temporal resolution needed to convey linguistic information is close to 40 ms. Since the average mora duration in the 60 sentences utilized here was 120–150 ms, this indicates that one Japanese mora, typically corresponding to one consonant and one vowel in this order (“Kinkakuji” has 5 morae including a special mora for “n”), can be conveyed by 3–4 still (spectral) frames. It thus is possible to synthesize intelligible speech connecting still frames in time, as if they were Lego blocks.

In order to understand the mechanism of speech communication, it is of vital importance to determine how far the speech signal can be degraded in the temporal dimension. It should be one of the unavoidable steps to examine how long homogeneous temporal units can be, still conveying linguistic information. To employ locally time-reversed speech systematically has been a substitute for this paradigm (e.g., Ueda et al., 2017). The reversed temporal units are never static, however, and this simply makes the interpretation of the perceptual data difficult. Mosaic speech whose frequency resolution was as fine as critical bands solved this problem; it was now established that static temporal units of 40 ms are sufficient for reasonable speech communication.

## 4. Materials and methods

### 4.1. Participants

Four (Experiment 1) or 20 (Experiment 2) native-Japanese speakers participated. They were 4 men of 21–25 years old (Experiment 1) or 9 women and 11 men of 19–25 years old (Experiment 2), all with normal hearing.

### 4.2. Stimuli

Four stimulus types were generated: (1) original speech with a 5-ms rise and a 5-ms fall time (Figure 2B), (2) mosaic speech (Figure 3B), (3) locally time-reversed speech with a 5-ms rise and a 5-ms fall time (Figure 4A), and (4) locally time-reversed speech without rise and fall times (Figure 4B). Only mosaic speech was employed in Experiment 1, while all four types of stimuli were employed in Experiment 2.

Japanese speech samples were obtained from the “NTT-AT Multi-Lingual Speech Database 2002.” The samples were spoken by a female native-Japanese speaker, and with a sampling rate of 16 kHz with 16-bit quantization. The samples were edited to remove irrelevant silent portions and noises, and converted into computer-oriented audio (.wav) files with a sampling frequency of 22.05 kHz using Praat (Boersma and Weenink, 2016).

Original-speech stimuli were shaped with a temporal grid as in the other types of stimuli. The width of the grid window was varied in 5 steps, 20, 40, 80, 160, and 320 ms, and the window edges were smoothed with 5-ms rise and fall times.

Mosaic speech stimuli were created by calculating the average sound-energy density within each spectrographic block of a speech sample. The speech signals were first delimited by narrow frequency bands, and then shaped by temporal windows with 5-ms rise and fall times, turning into mosaic speech. The width of each frequency band was determined so as to simulate a critical band in the same frequency range (Fastl and Zwicker, 2007). Seventeen frequency bands covering the range 0.1–4.4 kHz were utilized. This frequency range was enough to make noise-vocoded speech almost perfectly intelligible (Shannon et al., 1998). How sound energy density should change in time in each frequency band was calculated as a target, utilizing a moving average of intensity with a Gaussian window in time (σ = 5 ms). To realize this target, we generated a white noise as long as the speech signal, adding temporal margins. This noise was divided into the same 17 frequency bands. Sound energy density of this noise as a function of time was calculated from each frequency band utilizing the same moving average as above. This indicates an unavoidable small level fluctuation of the noise in each frequency band, which was going to be canceled to a certain degree in the next step. Finally, the noise within each frequency band was amplitude-modulated so that the original sound energy density of the noise was transformed to the calculated target density. Thus, the original speech was converted into a combination of band noises whose intensities were nearly constant within each time window, but with 5-ms rise and fall times. Locally time-reversed speech stimuli were shaped with a temporal grid of which the waveform in each temporal window was reversed in time, with and without 5-ms rise and fall times.

### 4.3. Conditions

In Experiment 1, only mosaic speech was used, and the grid window was varied in 4 steps. Three different speech sentences of 17–20 morae within the duration range of 2.27–2.98 s were used. Each participant encountered 3 sentences for each stimulus condition, adding up to 55–58 morae in total. The average duration of one mora calculated for each sentence was 0.13–0.15 s.

In Experiment 2, combining the 4 stimulus types and the 5 steps of the temporal grid resulted in 20 stimulus conditions in total. For each stimulus condition, 3 different speech sentences of 16–19 morae within the duration range of 2.13–2.66 s were used, adding up to 53 or 54 morae in total. Each participant thus encountered 60 stimuli in total. The average duration of one mora calculated for each sentence was 0.12-0.15 s.

In both experiments, each sentence appeared only once for each participant and for each stimulus condition. The sound energy per unit time of the speech stimuli was equalized.

### 4.4. Procedure

Each stimulus was presented once, diotically through headphones, 0.4 s after the participant clicked a “Play” button on the computer screen. After presentation, the participant wrote down on paper the morae he/she had heard in Japanese hiragana letters, avoiding guessing from the context as far as possible. A hiragana letter in most cases corresponds to a clearly distinguishable speech sound, but in some cases it was necessary for the participant to use both a hiragana letter and a few Roman letters to avoid ambiguity. How to do this was instructed clearly to the participant before the training trials. The 60 speech stimuli were randomly presented to each participant in 4 blocks. A warm-up trial was added to the 15 trials (stimuli) for each block. The participant was first asked to perform a practice block consisting of 20 trials. The sentences used in the practice block and the warm-up trials were not used again.

The research was conducted with prior approval of the Ethics Committee of Kyushu University; all methods employed were in accordance with the guidelines provided by the Japanese Psychological Association. The participants provided written informed consent prior to their participation.

The stimuli were presented to the participant in a soundproof room, from a computer (Frontier KZFM71/N) with an audio board (E-MU 0404) that was installed outside the soundproof room. From the computer, the stimuli were passed through an audio processor (Onkyo SE-U55GX), a low-pass filter (NF DV-04 DV8FL; cut-off frequency 15 kHz), a graphic equalizer (Roland RDQ-2031), and a headphone amplifier (STAX SRM-3235), before being presented to the participant through headphones (STAX SR-307). The equalizer was used to keep the flat shape of the frequency characteristics of the headphones, while the low-pass filter was used for anti-aliasing. Since the sampling frequency of the speech files was 22.05 kHz, the cut-off frequency of 15 kHz could not suitably deal with aliasing in between 14.05 and 15 kHz. The sound energy related to this range, however, was negligible. The audio output level was calibrated so that a 1-kHz pure tone of the same intensity as the average intensity of the stimuli was at 70 dBA with a precision sound level meter (Naganokeiki 2071) mounted with an artificial ear (Brüel and Kjær 4153).

## Author contributions

YN: designed the study, wrote prototypes of computer programs, analyzed the data, and wrote the paper; MM: designed the study, and collected and analyzed the data; KU and GR: prepared for the experiment, analyzed the data, and wrote the paper.

### Conflict of interest statement

The authors declare that the research was conducted in the absence of any commercial or financial relationships that could be construed as a potential conflict of interest.
