# Supplementary material for: Temporal Resolution Needed for Auditory Communication: Measurement With Mosaic Speech
Source: Front Hum Neurosci. 2018 Apr 24;12:149. doi: 10.3389/fnhum.2018.00149 (PMC5928238; doi:10.3389/fnhum.2018.00149)
Supplement: Supplementary file 1 [file Table_1.pdf]

Supplementary Material: Temporal Resolution Needed for Auditory Communication: Measurement with Mosaic Speech

1 SUPPLEMENTARY TABLE

Table S1. The results of a preliminary experiment with Japanese time-and-frequency-reversed speech.

	Temporal window width (ms)		
Categorized intelligibility (%)	40	80	160
100	10	0	0
< 100	4	0	0
< 75	7	0	0
< 50	0	0	0
< 25	1	22	22
Total number of participants	22	22	22
Averaged intelligibility (%)	78	13	4

Japanese spoken sentences, similar to those used in the main experiment, were converted to time-and-frequency-reversed speech. Eighteen narrow frequency bands simulating critical bands between 50 and 4,800 Hz were utilized so that time-frequency windows of (40, 80, or 160 ms) × (1 critical bandwidth) were obtained. One sentence of 18–20 morae was assigned to each condition for each participant. Obtained intelligibility scores of all the 22 participants in each condition, determined by the temporal window width, were calculated as a percentage of correct mora identification. The frequency distribution of the intelligibility scores in terms of numbers of participants is indicated in this table. As the temporal window width became wider than 40 ms, intelligibility decreased sharply.
